# Muscle-Derived Small Extracellular Vesicles Regulate Bone Maintenance During Hibernation Through miRNA-Mediated Signaling

**DOI:** 10.3390/cells15161468

**Published:** 2026-08-16

**Authors:** Yue He, Fangyang Pan, Yong Kong, Ziyi Zhang, Anni Wang, Mu Cui, Yuhong Niu, Yuan Gao, Kai Dang, Yongai Zhang

**Affiliations:** 1 School of Nursing and Rehabilitation, Xi’an Medical University, Xi’an 710021, China; hy@xiyi.edu.cn (Y.H.);; 2 Key Laboratory of Resource Biology and Biotechnology in Western China, Ministry of Education, Northwest University, Xi’an 710069, China; 3 Research Center for Special Medicine and Health Systems Engineering, School of Life Sciences, Northwestern Polytechnical University, Xi’an 710129, China

**Keywords:** hibernation, muscle–bone interaction, small extracellular vesicles, microRNA

## Abstract

**Highlights:**

**What are the main findings?**

- Small extracellular vesicles derived from the muscle (Mu-EVs) of hibernating ground squirrels may exert pro-osteogenic effects that modulate bone metabolism under prolonged disuse.
- A downregulated novel miRNA in TOR-Mu-EVs may partially relieve BMP7 suppression and activate Smad1/5/8 signaling, potentially sustaining bone homeostasis during hibernation.

**What are the implications of the main findings?**

- This study identifies a Mu-EV–miRNA regulatory axis as one of the potential preliminary mechanisms by which hibernators resist disuse osteoporosis.
- The miRNA/BMP7/Smad signaling pathway holds potential for identifying candidate molecular targets for treating disuse-mediated bone loss.

**Abstract:**

Prolonged skeletal muscle disuse, such as extended inactivity and mechanical unloading, typically elicits severe muscle atrophy and progressive bone loss, yet hibernating mammals evade this pathological cascade via poorly defined adaptive mechanisms. Using the Daurian ground squirrel (*Spermophilus dauricus*) as a unique natural model of prolonged torpor, we demonstrate that skeletal muscle-derived small extracellular vesicles (Mu-EVs) orchestrate protective muscle–bone crosstalk to maintain bone homeostasis during extended disuse. Morphological and microstructural analyses revealed no significant deficits in skeletal muscle and tibial bone between pre-hibernation (PRE) and torpor (TOR) states. Compared with PRE-Mu-EVs, TOR-Mu-EVs significantly enhanced osteogenic differentiation in MC3T3-E1 osteoblasts, markedly upregulating mRNA expression of the key osteogenic markers OCN and COL1A1 (*p* < 0.05, *p* < 0.01). Small RNA sequencing identified a novel unannotated miRNA (mature sequence: GCAGCAGCCCGGCTCTCCTAAT) sharply downregulated in TOR-Mu-EVs (*p* < 0.01); this miRNA exhibits binding potential toward the transcript of Bmp7, a pivotal regulator of osteogenesis. In vitro functional assays confirmed that this miRNA suppresses osteoblast maturation; in a mouse hindlimb unloading (HLU) disuse osteoporosis model, miRNA antagomir partially alleviated bone loss, boosting Masson staining area by 27.13% (*p* < 0.05) and bone volume fraction by 15.01% (*n* = 5, 0.05 < *p* < 0.1, Cohen’s d = 0.71, 95% CI [−0.16, 1.38]). Collectively, hibernating Mu-EVs mitigate this BMP7-inhibiting miRNA to sustain osteogenic activity, hinting at a conserved regulatory cascade that could offer tentative translational clues for managing disuse osteoporosis.

## 1. Introduction

Skeletal unloading caused by inactivity, immobilization, or microgravity rapidly induces muscle atrophy and bone demineralization, while concurrently increasing the risk of metabolic dysregulation, collectively diminishing physical function and quality of life [1,2]. Mitigating disuse-related musculoskeletal deterioration has therefore emerged as a critical goal across biomedical disciplines. In stark contrast to this pathological trajectory, hibernating mammals exhibit a remarkable physiological resilience. Despite enduring prolonged torpor accompanied by minimal movement and sustained neuromuscular inactivity, species such as the brown bear (*Ursus arctos*), Asiatic black bear (*Ursus thibetanus japonicus*), Daurian ground squirrel (*Spermophilus dauricus*), and golden-mantled ground squirrel (*Spermophilus lateralis*) display complete locomotor recovery upon arousal, with no evidence of appreciable muscle wasting or net skeletal loss [3,4,5,6,7]. These animals thus offer a unique, naturally occurring model for intrinsic resistance to disuse-induced musculoskeletal degeneration.

Conventional paradigms have emphasized that muscle–bone interactions are predominantly mediated by mechanical loading. Notably, skeletal muscle produces contractile forces that load the bone matrix, stimulating osteogenesis and contributing to the preservation of mineral density and mechanical strength. Bone, in turn, provides the rigid structural anchor necessary for force transmission, thereby establishing the upper limit of skeletal muscle hypertrophy [8,9]. However, growing evidence has expanded this view, revealing a biochemical signaling axis through which skeletal muscle exerts paracrine and endocrine control over skeletal remodeling. Myokines, osteokines, and muscle-derived exosomal cargoes—including microRNAs (miRNAs), proteins, and metabolites—circulate through interstitial and systemic pathways, epigenetically modulating osteoblast commitment, osteoclast differentiation, and osteocyte mechanotransduction [10,11,12,13]. Together, these findings support a model in which muscle–bone communication is governed by bidirectional mechanochemical crosstalk that synchronizes architectural remodeling with functional adaptation.

Small extracellular vesicles (sEVs), particularly exosomes, function as critical mediators of intercellular signaling, enabling molecular exchange between diverse cell types and organs [14]. Skeletal muscle-derived small extracellular vesicles (Mu-EVs) have attracted increasing attention for their capacity to stimulate myocyte proliferation, enhance muscle regeneration, and promote bone repair [15,16,17], indicating therapeutic potential in musculoskeletal dysfunction. As part of their bioactive cargo, miRNAs are especially notable for their evolutionary conservation and extensive regulatory effects on downstream targets. Multiple studies have shown that Mu-EVs regulate osteogenesis through miRNA-dependent signaling. C2C12-derived sEVs deliver miR-27a-3p to MC3T3-E1 preosteoblasts, promoting their differentiation via activation of the β-catenin pathway [18]. These sEVs also suppress osteoclast formation and enhance osteoblast function, primarily through high expression of miR-196a-5p [19]. In addition, sEVs containing miR-128 activate the YAP signaling pathway, accelerating osteogenic differentiation from bone marrow mesenchymal stem cells [20]. These findings demonstrate that Mu-EVs contribute to both bone formation and remodeling, supporting their role as molecular mediators through which skeletal muscle influences bone architecture and tissue maintenance.

In addition, previous research has illustrated that skeletal muscle-derived small extracellular vesicles under disuse conditions suppress osteogenic activity and accelerate bone resorption, ultimately driving bone loss [21]. Nevertheless, the functional properties of Mu-EVs in hibernators experiencing disuse remain poorly defined. Specifically, it remains unclear whether Mu-EVs miRNA profiles shift during prolonged inactivity, and whether such alterations contribute to skeletal preservation in hibernating species.

This study examined whether Mu-EVs derived from Daurian ground squirrels affect tibial bone morphology and osteogenesis during hibernation. Pre-hibernation and torpor-stage Mu-EVs were compared to assess differential miRNA cargo and to investigate the regulatory impact of specific miRNAs on osteogenic processes. By leveraging the remarkable resistance of hibernators to disuse-induced musculoskeletal decline, this work explored sEV-mediated signaling pathways that support skeletal preservation during extended inactivity. Given the recognition of sEVs as systemic endocrine messengers, defining their role in muscle–bone communication during hibernation may offer molecular insight into disuse resistance and inform strategies to prevent musculoskeletal deterioration.

## 2. Materials and Methods

### 2.1. Animals and Experimental Groups

Adult Daurian ground squirrels were captured between July and August 2024 in Weinan City, Shaanxi Province. All animal procedures were approved by the Experimental Ethics Committee of Xi’an Medical University (Approval No.: XYLS2025157). Following capture, the squirrels (weighing 200 ± 20g) were housed under semi-free-range conditions at the local feeding base. In early October, they were transferred to the university’s animal breeding room and maintained at approximately 25 °C with free access to food and water. Based on seasonal and physiological states (Figure S1 and Table S1), animals were allocated to the pre-hibernation (PRE) and torpor (TOR) groups (*n* = 6 in each group, all males). The PRE group was maintained under natural light–dark cycles and sampled in mid-to-late October. The TOR group underwent induced hibernation beginning in mid-to-late November in a dark hibernation chamber (4–8 °C) and was sampled in early January.

### 2.2. Sample Collection

The squirrels were anesthetized via intraperitoneal injection of pentobarbital sodium (45 mg/kg). The soleus, extensor digitorum longus, and tibia were excised, weighed, and processed for downstream analyses. Following sample collection, animals were euthanized via administration of an overdose of anesthetic and disposed of according to institutional biosafety protocols.

### 2.3. Cryosectioning

Muscle samples were embedded in OCT compound and mounted on a cryostat (4583, Sakura Finetek, Torrance, CA, USA). Sectioning was performed at −25 °C, producing 50 μm thick transverse sections perpendicular to the muscle fiber axis. Sections were affixed to anti-slip glass slides, dried in an oven at 37 °C for 30 min, and stored at −20 °C for future use.

### 2.4. Immunofluorescence Staining

Slides were equilibrated to room temperature and incubated in PBS for 10 min, followed by fixation in 4% paraformaldehyde for 30 min. After rinsing with PBS, tissues were permeabilized in PBS containing 10% bovine serum albumin (BSA) and 1% Triton X-100 for 1 h, then washed three times with PBS (10 min each). Blocking was performed with 7.5% BSA (20 μL per section) for 1 h at room temperature. Slides were then incubated overnight at 4 °C with primary antibody against Laminin (1:400; BA1761-1, Boster, Wuhan, China) diluted in 4% BSA. After washing, sections were incubated for 2 h at 37 °C with Alexa Fluor 647-conjugated secondary antibody (1:300; A-21235, Thermo Fisher, Waltham, MA, USA). After further PBS washes (3 × 10 min), sections were mounted with antifade medium (G1401-25ML, Servicebio, Wuhan, China). Imaging was performed under 40× magnification using a laser confocal microscope (TCS SP8, Leica, Wetzlar, Germany). For each sample, five fields were acquired using a cross-diagonal sampling approach, resulting in 15 images per animal. Cross-sectional areas of individual fibers were quantified from laminin-stained contours using Image-Pro Plus v6.0. After repeating this procedure for all six animals in each group, group-level means were calculated and reported as the fiber cross-sectional area.

### 2.5. Micro-CT Detection

After sample collection, high-resolution imaging of the tibia was performed using a Micro-CT scanner (Quantum GX2, PerkinElmer, Waltham, MA, USA) under the following parameters: X-ray tube voltage, 50 kVp; current, 80 μA; filter combination: Cu 0.06 mm + Al 0.5 mm. The region of interest (ROI) was defined relative to the lowest point of the distal tibial growth plate, with a 2.0 mm segment beginning 0.5 mm distal to this reference point. Within this segment, 100 consecutive slices were acquired at a voxel size of 20 μm. ROI segmentation was conducted using a combined manual and threshold-based approach to distinguish cortical and trabecular compartments. Quantitative parameters were extracted, including cortical area (Ct.Ar, mm^2^), trabecular number (Tb.N, 1/mm), cortical bone thickness (Ct.Th, mm), and bone volume fraction (BV/TV, %).

### 2.6. Isolation and Culture of Primary Skeletal Muscle Cells

Animals were anesthetized via intraperitoneal injection of sodium pentobarbital (45 mg/kg). The hind limbs were disinfected in 75% ethanol, and the soleus muscle was excised under sterile conditions. Muscle samples were rinsed three times with Hanks’ balanced salt solution (HBSS) supplemented with 5% antibiotic–antimycotic solution (penicillin 100 U/mL, streptomycin 100 μg/mL; Beyotime Biotechnology Institute, Nanjing, China). Adipose and connective tissues were removed, and the muscle was minced into 0.1 cm^3^ fragments. The minced tissue was transferred into 15 mL sterile centrifuge tubes, washed three times with HBSS, and centrifuged (1000 rpm, 5 min) to discard the supernatant. Digestion was performed in 2 mL of 0.2% type II collagenase (17101015; Gibco, Waltham, MA, USA) for 30 min at 37 °C in a 5% CO_2_ cell incubator (371; Thermo Fisher, Waltham, MA, USA). The reaction was terminated by adding Dulbecco’s Modified Eagle Medium (DMEM)/F12 (CGM104.05; CellMax, Beijing, China) containing 10% fetal bovine serum (FBS; SA201.01; CellMax, Beijing, China). Following centrifugation, cells were resuspended in fresh complete DMEM/F12 and seeded into T25 flasks (25 cm^2^), incubated at 37 °C with 5% CO_2_ for 1 h. Fibroblasts in the cell suspension were removed using differential adhesion: non-adherent cells were transferred to new flasks with fresh medium and returned to the incubator for continuous culture. At the third passage, the medium was replaced with exosome-depleted FBS-containing complete medium (iCell-0650; Cellverse, Shanghai, China). Supernatants were collected at 24 h intervals and stored at −80 °C until subsequent use.

### 2.7. Small Extracellular Vesicle (sEV) Isolation and Characterization

sEVs were isolated by ultracentrifugation (Optima XE; Beckman, Brea, CA, USA) using the following protocol. Frozen skeletal muscle cell supernatants were thawed on ice and centrifuged at 600× *g* for 10 min at 4 °C. The resulting supernatant was collected and transferred to new sterile 50 mL tubes and centrifuged at 3000× *g* for 15 min at 4 °C. After filtration through a 0.22 μm filter membrane, the supernatant was centrifuged at 110,000× *g* for 70 min at 4 °C. The sEV pellet was resuspended in 100 μL of pre-chilled sterile phosphate-buffered saline (PBS) and stored at −80 °C until further use. Morphological assessment was conducted by means of transmission electron microscopy (TEM), and clearly delineated exosomal vesicles were selected for imaging. Particle size distribution was analyzed using a nanoparticle tracking analyzer (NanoSight 300; Malvern Panalytical Ltd., Malvern, UK).

### 2.8. Western Blot Analysis

Tissue or cell samples were homogenized in RIPA lysis buffer on ice. Homogenates were centrifuged at 15,000 rpm for 15 min at 4 °C, and the resulting supernatant was collected. Protein concentration was determined using a protein assay kit (23227, Thermo Fisher Scientific, Waltham, MA, USA). Supernatants were mixed with loading buffer at a 1:4 (*v*/*v*) ratio to achieve a final protein concentration of 1.5 μg/μL. Samples were then boiled for 10 min and stored at −80 °C until analysis. Proteins were separated via sodium dodecyl sulfate-polyacrylamide gel electrophoresis (SDS-PAGE) using 10% polyacrylamide gels containing 0.5% trichloroethanol at a constant pressure of 120 V. Subsequently, the proteins were transferred to polyvinylidene fluoride (PVDF) membranes using a wet-transfer method, following the sequence of filter paper, gel, PVDF membrane, and filter paper to construct the transfer stack. Membranes were blocked with 5% BSA blocking buffer at room temperature for 2 h, followed by overnight incubation with primary antibodies at 4 °C. After three washes with Tris-buffered saline containing Tween-20 (TBST) for 10 min each, membranes were incubated with secondary antibodies for 2 h at room temperature. Following additional TBST washes, protein signals were visualized using a chemiluminescence reagent (NCI5079, Thermo Fisher Scientific, Waltham, MA, USA) and imaged with a gel documentation system (G-box; Cambridge, UK). Control group values were used as a normalization control for multiple plates. To ensure reliable normalization, total protein staining of the gel was used as an internal control. Densitometric analysis was performed, and relative protein expression was calculated as the ratio of target protein signal intensity to the total protein signal in the corresponding gel lane. Details of antibodies used are provided in Table S2.

### 2.9. Effect of Mu-EVs on Osteoblasts

Osteoblasts were seeded onto circular coverslips placed in 6-well plates at a density of 2 × 10^5^ cells/cm^2^. Each well received 50 μL of PKH67-labeled sEV solution, and cells were incubated at 37 °C in a 5% CO_2_ cell incubator for 24 h. After incubation, the culture medium was gently aspirated using a pipette, and cells were fixed with 4% paraformaldehyde for 20 min, followed by three PBS washes (3 min each). Nuclei were counterstained with 4’,6-diamidino-2-phenylindole (DAPI) for approximately 10 min, and excess stain was removed by three additional PBS washes (5 min each). Coverslips with adherent cells were then removed, and the intracellular distribution of sEVs was observed under a laser scanning confocal microscope (LSM; TCS SP8; Leica, Wetzlar, Germany).

MC3T3-E1 preosteoblasts were obtained from the Cell Bank of the Chinese Academy of Sciences. Cells were seeded into 6-well plates precoated with 0.1% gelatin (GLT-11301; Cyagen Biotechnology, Guangzhou, China) at a density of 2 × 10^4^ cells/cm^2^ and cultured to approximately 80% confluency. For the control group, 2 mL of osteogenic differentiation medium containing osteogenic induction solution (MUXMT-04021; Cyagen Biotechnology, Guangzhou, China) was added per well. Experimental groups received the same osteogenic medium supplemented with 200 μL of an sEV suspension containing either PRE-Mu-EVs or TOR-Mu-EVs at a protein concentration of 2.0 μg/μL. Cultures were maintained at 37 °C with medium replacement every 48 h. On day 14, cells were harvested for qPCR (mRNA) detection of osteogenic markers, including ALP, OCN, RUNX2, and Col1a1.

### 2.10. Quantitative Real-Time PCR (qPCR)

MC3T3-E1 cells were harvested at a density exceeding 1 × 10^6^ cells/mL. Total RNA was extracted using the TRIzol method based on guanidinium thiocyanate-phenol-chloroform phase separation. Complementary DNA (cDNA) was synthesized from the extracted RNA using a commercial reverse transcription kit (B639277-0050; Sangon Biotech, Shanghai, China) and diluted to the required concentration. The qPCR reactions were prepared using standard reagents in a 96-well format, with gene-specific primers provided in Table S3. Amplification was conducted on a LightCycler 96 system (Roche, Basel, Switzerland).

### 2.11. Omics Analysis of Mu-EV miRNA

Total RNA was isolated and purified from sEVs, and small RNA fractions were subjected to next-generation sequencing (NGS). Raw reads were processed to remove low-quality sequences, and clean reads were annotated via alignment to small RNA databases and the reference genome using Bowtie [22]. Known miRNAs were identified through comparison with the Rfam database using CMsearch [23]. Novel miRNAs were predicted using miRDeep2 [24]. All miRNAs were quantified based on mapped read counts, and differentially expressed (DE) miRNAs were screened using DESeq2 with thresholds of |log_2_(fold change)| ≥ 1 and adjusted *p* < 0.05. Target genes of DE-miRNAs were predicted jointly by RNAhybrid and miRanda [25,26]; only overlapping targets predicted by both algorithms were retained for subsequent functional enrichment analysis.

Relative expression levels of a representative novel miRNA (mature sequence: GCAGCAGCCCGGCTCTCCTAAT), screened by omics analysis, were validated by qPCR as described above, with the exception that stem-loop reverse transcription was performed. Relevant primers are provided in Table S4.

### 2.12. Dual-Luciferase Reporter Assay

Wild-type (WT) and mutant (MT) sequences of the mouse BMP7 gene were synthesized and cloned into the pmirGLO vector, which encodes firefly luciferase as the primary reporter and Renilla luciferase as an internal control (plasmid constructs detailed in Table S5). For transfection, mouse NIH3T3 cells were seeded into 24-well plates and incubated with 500 μL of transfection mixture comprising the constructed plasmid, synthesized miRNA, and Lipofectamine 2000 transfection reagent (11668-027; Invitrogen, Waltham, MA, USA) for 6 h at 37 °C. After aspirating the transfection complex, 1 mL of complete medium was added and the cells were incubated for an additional 48 h. Cells were then lysed with 200 μL of cell lysis buffer per well and shaken for 5 min. Lysates were centrifuged at 10,000 rpm at 4 °C for 5 min, and the supernatants were collected for luminescence measurement using a Dual-Luciferase Reporter Assay Kit (11402ES60; Yeasen Biotechnology, Shanghai, China). Luminescence intensity was measured at a wavelength range of 350–700 nm. Reporter activity was quantified by calculating the ratio of firefly luciferase to Renilla luciferase relative light units (RLU).

### 2.13. Transfection of MC3T3-E1 Cells

Synthetic agomirs (mimics), antagomirs (inhibitors), and negative control (NC) sequences targeting the novel miRNA were prepared (sequences are provided in Table S6). MC3T3-E1 cells were transfected using a mixture of 15 μL of transfection reagent and 2 μL of RNA per well. After 6 h of incubation, the transfection medium was removed and replaced with complete medium containing osteogenic induction solution (MUXMT-04021; Cyagen Biosciences, Suzhou, China). At 48 h post-transfection, total RNA was extracted for qPCR to quantify the mRNA expression of ALP, OCN, RUNX2, and BMP7 (primer information is provided in Table S3). At 96 h post-transfection, protein extracts were analyzed by Western blot analysis to determine the expression levels of OCN, OPN, RUNX2, BMP7, Smad1/5/8, and phosphorylated Smad1/5/8 (p-Smad1/5/8) (antibody information is provided in Table S7).

### 2.14. In Vivo Transfection

Male BALB/c mice (6 weeks old, body weight 20–22 g) were purchased from the Laboratory Animal Center of Xi’an Jiaotong University. A total of 20 mice were randomly assigned into four experimental groups (*n* = 5 per group). The disuse osteoporosis model was established via tail suspension intervention for a consecutive period of 28 days [27]. During the suspension period, experimental animals received twice-weekly intramuscular injections of the transfection mixture into the hindlimb gastrocnemius muscles at approximately 3-day intervals. Each injection contained 5 μL of nucleic acid, 0.6 μL of in vivo-jetPEI transfection reagent (101000040; Polyplus-transfection, Illkirch, France), and 5% glucose solution as the solvent. After 4 weeks, femurs were collected for weight measurement, micro-CT scanning, and Masson staining.

### 2.15. Masson Staining

Bone samples were decalcified using a commercial decalcifying agent (G1105; Servicebio Technology, Wuhan, China), embedded in paraffin, and sectioned. Sections were dewaxed with xylene and sequentially dehydrated through graded anhydrous ethanol. Hematoxylin staining was performed for 6 min, followed by rinsing in tap water and differentiation in acid ethanol for 15 s. Sections were subsequently immersed in ponceau–fuchsin staining solution for 5 min, rinsed with distilled water, treated with phosphomolybdic acid solution for 2 min and rinsed with 1% glacial acetic acid for 30 s. Aniline blue was applied for 5 min, followed by a final rinse in 1% glacial acetic acid for 1 min. Sections were then dehydrated in gradient ethanol, cleared in xylene, air-dried, and mounted with neutral balsam. Images were captured using an optical microscope (Nikon Eclipse C1; Nikon, Tokyo, Japan).

### 2.16. Statistical Analysis

All data are presented as mean ± standard deviation (SD). Statistical analyses were performed using SPSS Statistics v19 (IBM Corp., Armonk, NY, USA). The Shapiro–Wilk test was conducted to verify the normality. Homogeneity of variances was assessed using Levene’s test. One-way analysis of variance (ANOVA) with Tukey’s post hoc test was applied for multiple group comparisons when data satisfied both normality and homogeneity of variances (*p* > 0.05). The independent samples t-test was used for comparisons between two groups. *p* < 0.05 was considered statistically significant.

## 3. Results

### 3.1. Changes in Skeletal Muscle and Bone Mass and Morphology

**Figure 1:**
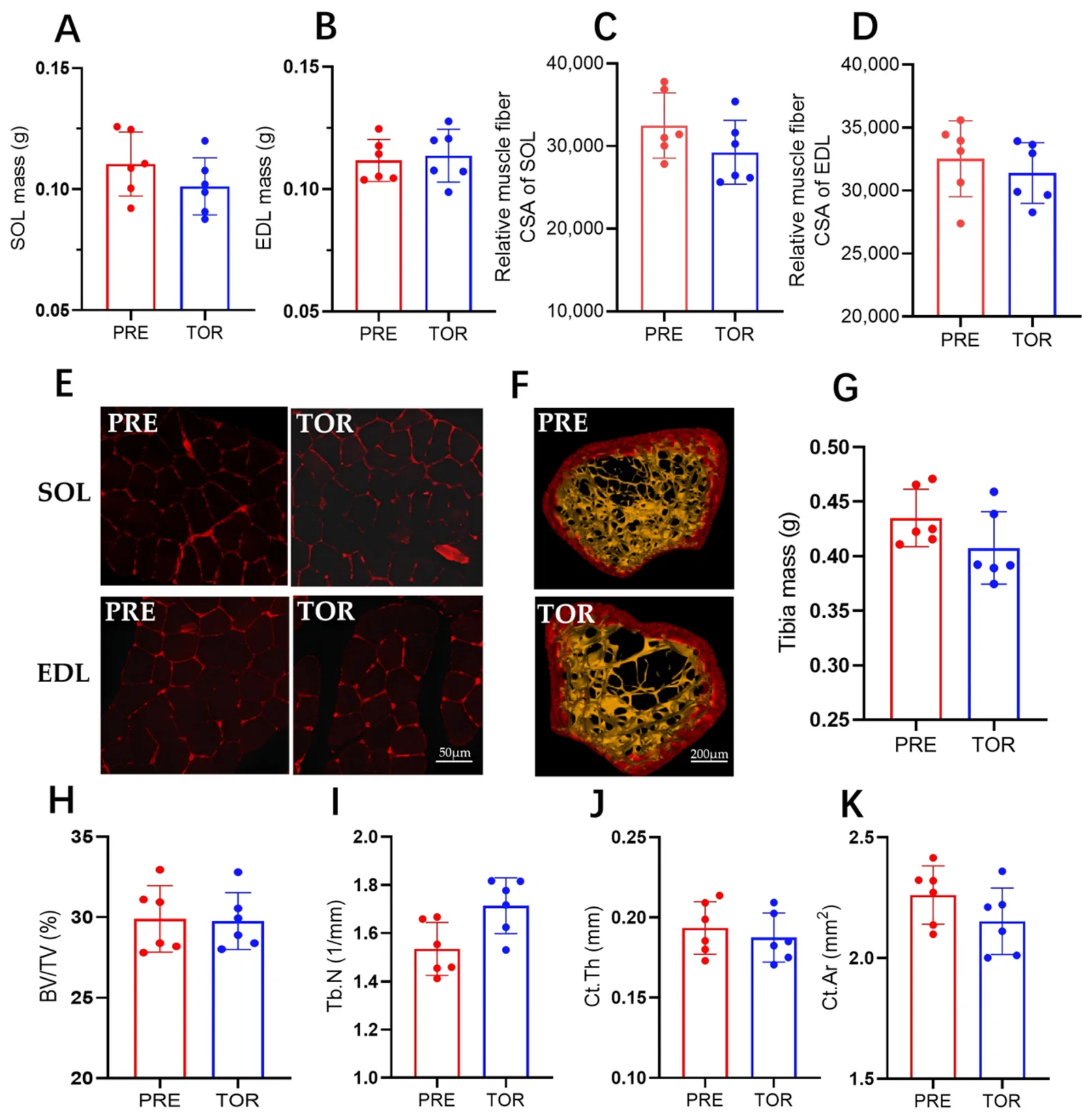
Changes in musculoskeletal mass and microstructure in different periods. (**A**) Changes in SOL mass between groups. (**B**) Changes in EDL mass between groups. (**C**) Changes in SOL fiber CSA between groups. (**D**) Changes in EDL fiber CSA between groups. (**E**) Immunofluorescence images (antibody: laminin) of SOL and EDL fiber CSA between groups (scale bar = 50 μm). (**F**) Representative micro-CT images of tibia between groups (scale bar = 200 μm). (**G**) Changes in tibia mass between groups. (**H**) Histogram of BV/TV between groups. (**I**) Histogram of Tb.N between groups. (**J**) Histogram of Ct.Th between groups. (**K**) Histogram of Ct.Ar between groups. Data were analyzed using *t*-test. Mean ± SD, *n* = 6. SOL, soleus; EDL, extensor digitorum longus; CSA, cross-sectional area; PRE, pre-hibernation group; TOR, torpor group. Ct.Th, cortical thickness; Tb.N, trabecular number; BV/TV, bone volume fraction; Ct.Ar, cortical bone area.

Compared with the pre-hibernation (PRE) group, soleus muscle mass exhibited no statistically significant differences in the torpor (TOR) group (*p* > 0.05, Figure 1A). Likewise, extensor digitorum longus (EDL) muscle mass was comparable between the PRE and TOR groups with no statistical significance (*p* > 0.05, Figure 1B), indicating similar disuse sensitivity between slow-twitch and fast-twitch muscle subtypes in hibernators. The cross-sectional area (CSA) of muscle fibers did not differ significantly in either muscle across groups (*p* > 0.05, Figure 1C–E).

No significant difference in tibia mass between the PRE group and the TOR group (*p* > 0.05, Figure 1G). Micro-computed tomography (micro-CT) analysis revealed no significant differences in bone volume fraction (BV/TV), trabecular number (Tb.N), cortical thickness (Ct.Th), or cortical area (Ct.Ar) across timepoints (Figure 1F,H–K), suggesting that overall bone microstructure was not markedly altered during hibernation.

### 3.2. Characterization of Mu-EVs

**Figure 2:**
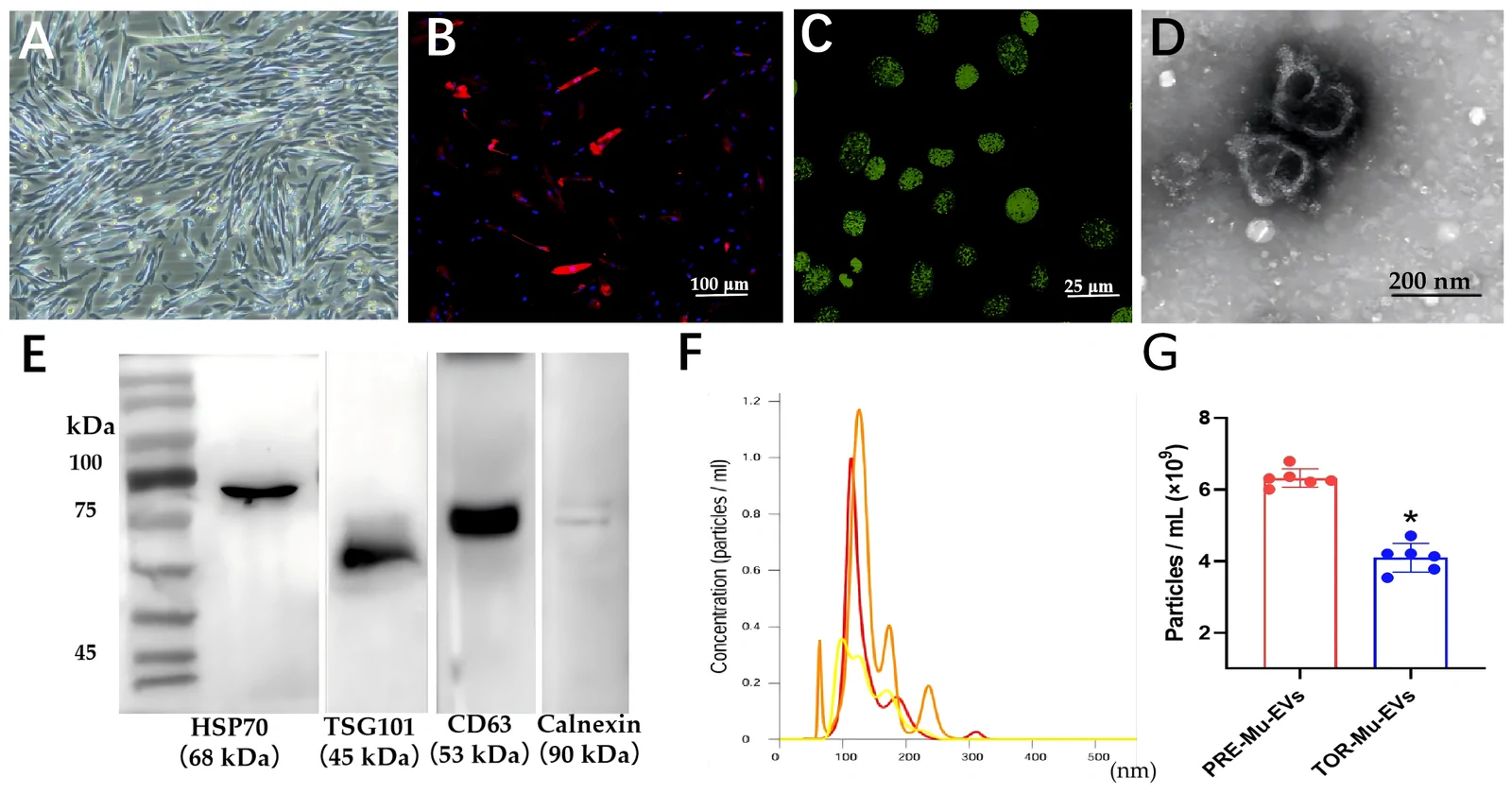
Culture of primary skeletal muscle myoblasts and identification of Mu-EVs. (**A**) Morphology of primary skeletal muscle myoblasts (40×). (**B**) DAPI (blue) and desmin (red) fluorescence staining of myoblasts (scale bar = 100 μm). (**C**) MyoD (green) fluorescence staining of myoblasts (scale bar = 25 μm). (**D**) Mu-EV morphology under transmission electron microscopy (scale bar = 200 nm). (**E**) Western blot detection of exosomal proteins. (**F**) Particle size distribution of Mu-EVs. (**G**) Comparison of Mu-EV concentration between pre-hibernation and torpor groups. HSP70, heat shock protein 70; TSG101, tumor susceptibility gene 101 protein; PRE-Mu-EVs, muscle-derived small extracellular vesicles during pre-hibernation; TOR-Mu-EVs, muscle-derived small extracellular vesicles during torpor. Data were analyzed using *t*-tests. Mean ± SD, *n* = 6; *, *p* < 0.05.

Primary skeletal muscle myoblasts adopted a spindle-shaped morphology upon adhesion (Figure 2A). Immunostaining for Desmin and MyoD confirmed their identity as myoblasts (Figure 2B,C). Mu-EVs were isolated via ultracentrifugation. Transmission electron microscopy (TEM) showed hemispherical or cup-shaped vesicles (Figure 2D). Western blotting revealed positive bands for the exosomal marker proteins HSP70, TSG101, and CD63, while no band was detected for the endoplasmic reticulum marker protein calnexin Figure 2E), with particle sizes predominantly ranging from 30 to 150 nm (Figure 2F), characteristic of sEVs. Furthermore, sEV concentration was significantly lower in the TOR group than in the PRE group (*p* < 0.05, Figure 2G).

### 3.3. Effect of Mu-EVs on Osteoblasts

**Figure 3:**
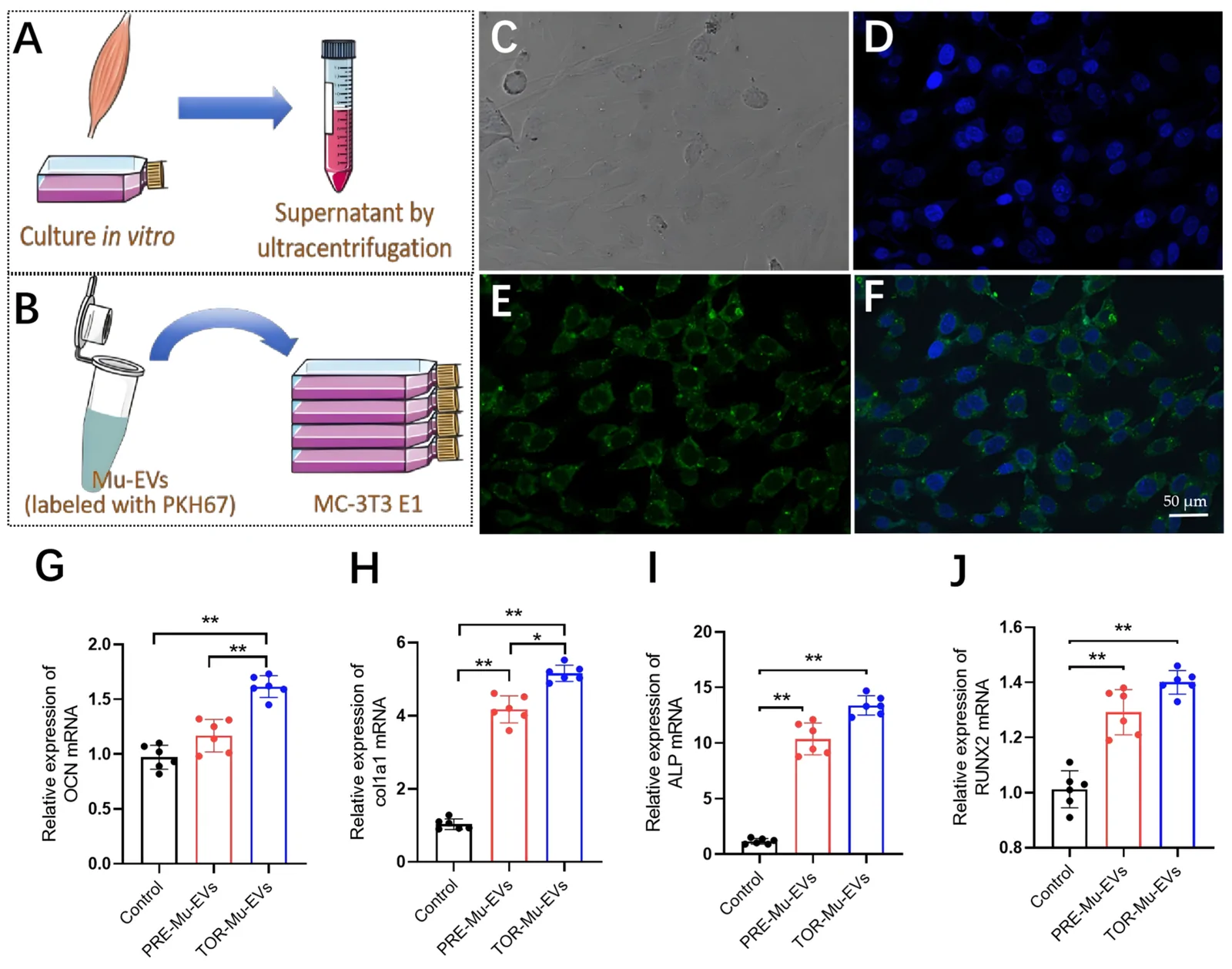
Muscle-derived small extracellular vesicles (Mu-EVs) co-cultured with MC3T3-E1 promote osteogenesis. (**A**) Skeletal muscle was cultured in vitro, and Mu-EVs were isolated by ultracentrifugation. (**B**) Co-culture of PKH67-labeled Mu-EVs with MC3T3-E1 cells. (**C**) Bright-field image of MC3T3-E1 cells. (**D**) DAPI-stained nuclei of MC3T3-E1 (blue fluorescence). (**E**) PKH67-labeled muscle-derived small extracellular vesicles (green fluorescence). (**F**) Merged image showing uptake of muscle-derived small extracellular vesicles by MC3T3-E1 cells (surrounding the cell nucleus) (scale bar = 50 μm). (**G**) Quantification of OCN mRNA expression in MC3T3-E1 cells induced by muscle-derived small extracellular vesicles at different stages. (**H**–**J**) Quantification of COL1a1, ALP, and Runx2 mRNA expression, respectively. Data were analyzed using ANOVA with post hoc Tukey’s test. Mean ± SD, *n* = 6, *, *p* < 0.05; **, *p* < 0.01. Control, control group; sEVs, small extracellular vesicles; PRE-Mu-EVs, muscle-derived small extracellular vesicles during pre-hibernation; TOR-Mu-EVs, muscle-derived small extracellular vesicles during torpor. ALP, alkaline phosphatase; OCN, osteocalcin; COL1a1, collagen type I alpha 1 chain; Runx2, Runt-associated transcription factor 2.

MC3T3-E1 preosteoblasts were co-cultured with sEVs derived from pre-hibernation (PRE-Mu-EVs) or torpor (TOR-Mu-EVs) skeletal muscle. Fluorescence tracing revealed that PKH67-labeled Mu-EVs were internalized by preosteoblasts and localized to the cytoplasm, where they displayed a characteristic perinuclear aggregation pattern (Figure 3A–F). Quantitative real-time polymerase chain reaction (qPCR) results showed that mRNA levels of COL1A1, ALP, and RUNX2 were significantly elevated in both PRE-Mu-EVs and TOR-Mu-EVs compared to untreated controls (Figure 3H–J; *p* < 0.01). Furthermore, TOR-Mu-EVs induced significantly higher expression of OCN and COL1A1 compared to PRE-Mu-EVs (Figure 3G, H; *p* < 0.05 and *p* < 0.01, respectively), indicating that TOR-Mu-EVs exert stronger inductive effects on genes associated with mid-to-late osteogenic differentiation relative to PRE-Mu-EVs.

### 3.4. miRNA Profiling of Mu-EVs and Validation of Target Genes

**Figure 4:**
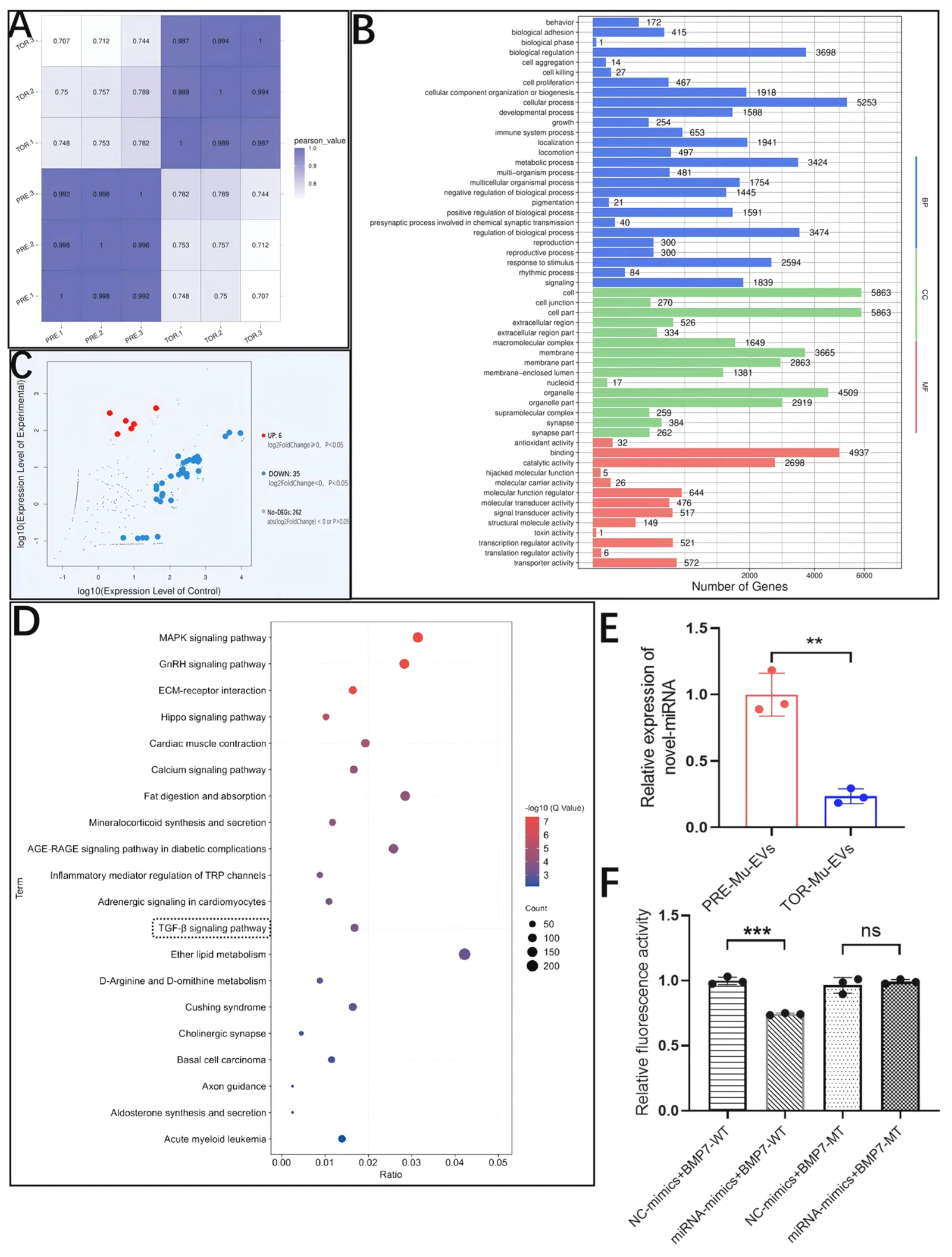
Omics analysis of miRNA in muscle-derived small extracellular vesicles and identification of target genes. (**A**) Heatmap of Pearson correlation coefficients between PRE-Mu-EVs and TOR-Mu-EVs (higher values indicate greater sample similarity). (**B**) GO functional classification of predicted target genes of differentially expressed miRNAs (BP, biological process; CC, cellular component; MF, molecular function). (**C**) Distribution of differentially expressed miRNAs (blue: significant downregulation; red: significant upregulation). (**D**) KEGG enrichment analysis of target genes of differentially expressed miRNAs. (**E**) Expression level of the novel miRNA in PRE-Mu-EVs and TOR-Mu-EVs. (**F**) Identification of BMP7 as a direct target of the novel miRNA using dual-luciferase reporter assay. Data were analyzed using ANOVA with post hoc Tukey’s test. Mean ± SD, *n* = 3; **, *p* <0.01; ***, *p* < 0.001. PRE-Mu-EVs, muscle-derived small extracellular vesicles during pre-hibernation; TOR-Mu-EVs, muscle-derived small extracellular vesicles during torpor; BMP7, bone morphogenetic protein 7; WT, wild-type; MT, mutant type.

High correlation coefficients were observed among nucleic acid samples from both groups, indicating overall consistency in miRNA expression profiles within each condition (Figure 4A). Despite this similarity, differential miRNA analysis identified distinct miRNA cargo composition between PRE-Mu-EVs and TOR-Mu-EVs. Compared with the PRE group (control), the TOR group exhibited six significantly upregulated and 35 significantly downregulated miRNAs (Figure 4C). Gene Ontology (GO) analysis of predicted miRNA targets revealed enrichment in fundamental biological processes, including metabolism, cellular function, and regulation. Notably, cellular component terms were primarily associated with cells, cell parts, and organelles, while molecular function terms were dominated by binding interactions and catalytic activity (Figure 4B). Kyoto Encyclopedia of Genes and Genomes (KEGG) enrichment analysis further linked these targets to multiple signaling pathways, including TGF-β signaling pathway, MAPK signaling pathway, ECM–receptor interaction, and ether lipid metabolism (Figure 4D). Together, these patterns associate Mu-EV miRNA cargo during hibernation with signaling networks relevant to musculoskeletal regulation.

miRNAs with potential osteogenic function were prioritized. A novel miRNA (sequence: GCAGCAGCCCGGCTCTCCTAAT) was screened according to the following criteria: fold change > 1.5 and mapped reads ≥ 10. The target prediction criteria comprised ≥6mer binding sites (TargetScan) and mfe ≤ −20 kcal/mol (RNAhybrid). Its predicted target, BMP7 (belong to the TGF-β signaling pathway), was selected for experimental validation. Notably, sequencing coverage for this candidate miRNA is limited (a total of 32 reads), which restricts the calculation of certain conservation-based metrics within miRDeep2. qPCR analysis confirmed significantly lower expression of this miRNA in TOR-Mu-EVs compared to PRE-Mu-EVs (Figure 4E; *p* < 0.01), consistent with sequencing data. Dual-luciferase reporter assays showed that co-transfection of miRNA mimics with the BMP7 wild-type (WT) construct significantly reduced luciferase activity relative to the NC-mimics + BMP7-WT control (*p* < 0.001; Figure 4F). No significant differences were observed among the NC-mimics + BMP7-WT, NC-mimics + BMP7-MT, and novel miRNA-mimics + BMP7-MT groups (*p* > 0.05), confirming specific interaction between the novel miRNA and BMP7 mRNA.

### 3.5. Effects of Novel miRNA on Osteogenic Differentiation in MC3T3-E1 Cells

**Figure 5:**
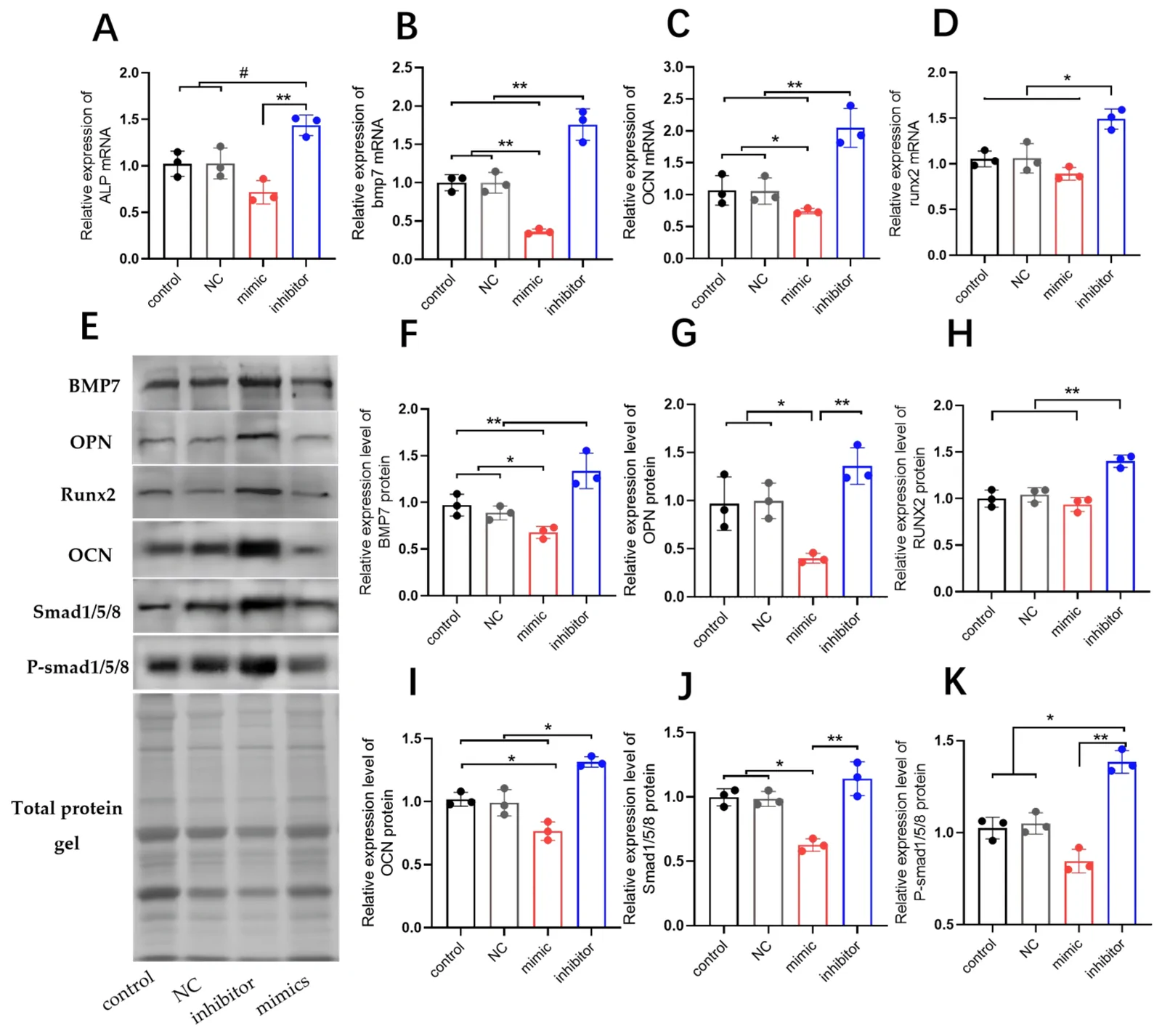
Effect of miRNA transfection on osteogenesis of MC3T3-E1 cells. (**A**) Histogram of relative expression of ALP mRNA 48 h post-transfection. (**B**) Histogram of relative expression of BMP7 mRNA 48 h post-transfection. (**C**) Histogram of relative expression of OCN mRNA 48 h post-transfection. (**D**) Histogram of relative expression of runx2 mRNA 48 h post-transfection. (**E**) Representative Western blot images at 96 h post-transfection. (**F**) Histogram of relative expression level of BMP7 96 h post-transfection. (**G**) Histogram of relative expression of OPN protein 96 h post-transfection. (**H**) Histogram of relative expression of RUNX2 protein 96 h post-transfection. (**I**) Histogram of relative expression level of OCN 96 h post-transfection. (**J**) Histogram of relative expression of Smad1/5/8 96 h post-transfection. (**K**) Histogram of relative expression of p-Smad1/5/8 protein 96 h post-transfection. Data were analyzed using ANOVA with post hoc Tukey’s test. Mean ± SD, *n* = 3; #, 0.05 < *p* < 0.1; *, *p* < 0.05; **, *p* < 0.01. control, blank control group; NC, negative control; mimics, novel-miRNA agomir group; inhibitor, novel-miRNA antagomir group. ALP, alkaline phosphatase; BMP7, bone morphogenetic protein 7; OCN, osteocalcin; RUNX2, runt-associated transcription factor 2; p-Smad1/5/8, phosphorylated-Smad1/5/8.

At 48 h post-transfection, ALP mRNA levels increased by 50.11% in the novel-miRNA inhibitor group compared to the control, showing a trend toward (0.05 < *p* < 0.1), while no significant differences was observed in the mimics group (*p* > 0.05, Figure 5A). Inhibitor treatment significantly elevated BMP7, OCN, and RUNX2 mRNA expression relative to both the control and negative control (NC) groups. In contrast, BMP7 and OCN mRNA levels were significantly reduced in the mimics group compared to the controls (Figure 5B–D; *p* < 0.01 and *p* < 0.05, respectively).

At 96 h post-transfection, protein levels of BMP7, OCN, RUNX2, and phosphorylated Smad1/5/8 (p-Smad1/5/8) were significantly upregulated in the inhibitor group compared to both the NC and mimics groups (Figure 5F,I,H,K; *p* < 0.05 or *p* < 0.01). Conversely, BMP7, OPN, OCN, and Smad1/5/8 protein levels were significantly lower in the mimics group compared to controls (Figure 5F,G,I,J; *p* < 0.05). A decreasing trend in p-Smad1/5/8 protein expression was also observed in the mimics group (Figure 5K; 0.05 < *p* < 0.1).

### 3.6. Effects of Novel miRNA on Bone in Mice Following Hindlimb Unloading (HLU)

**Figure 6:**
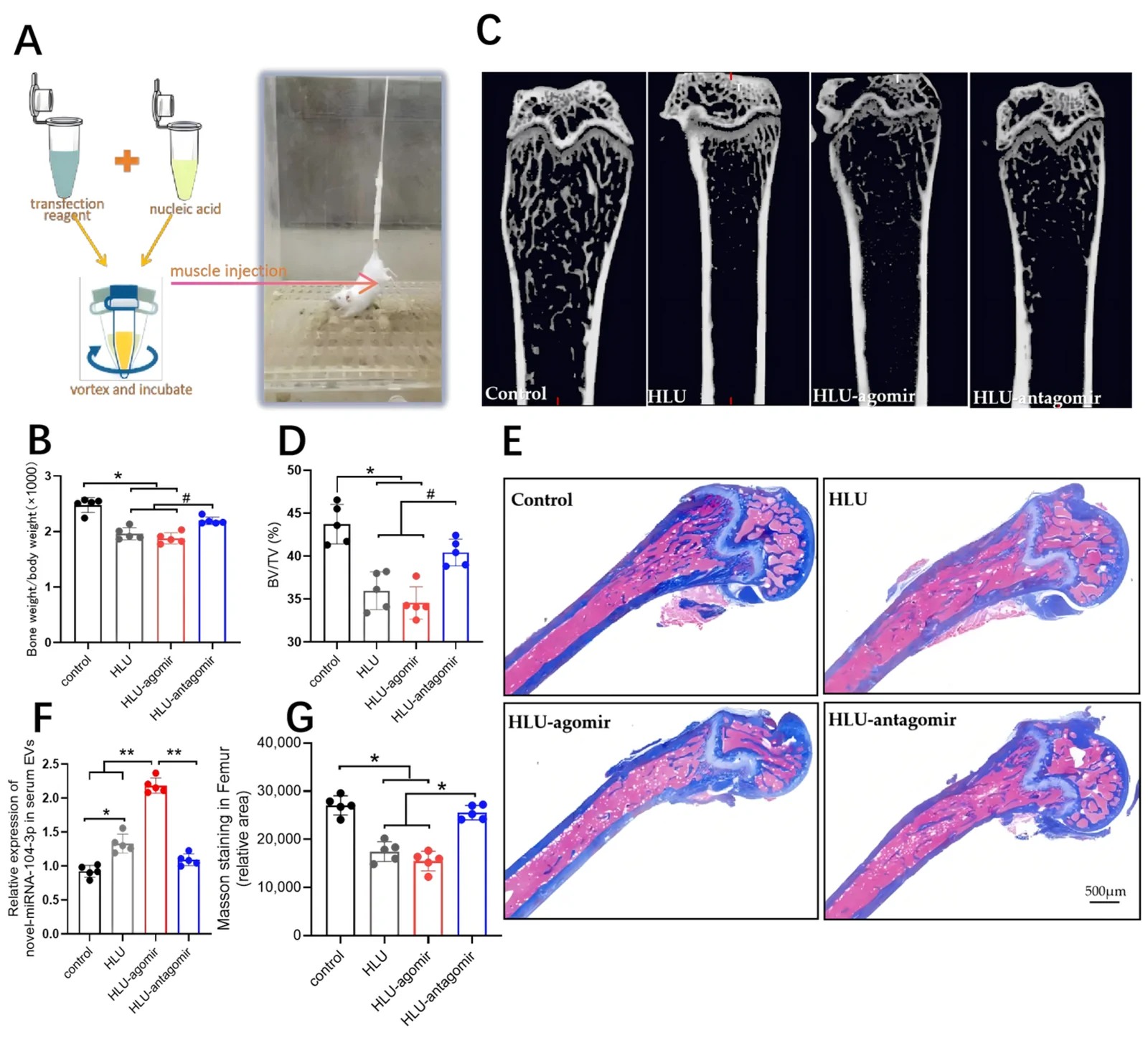
Effects of in vivo miRNA transfection on femoral bone in HLU mice. (**A**) Schematic of the HLU model (disuse osteoporosis) and intramuscular administration protocol. (**B**) Histogram of femur mass across experimental groups. (**C**) Representative micro-CT images of femurs. (**D**) Histogram of BV/TV. (**E**) Representative Masson-stained femoral sections (scale bar = 200 μm). (**F**) Histogram of serum exosomal novel miRNA content in each group. (**G**) Quantification of Masson staining results. Data were analyzed using ANOVA with post hoc Tukey’s test. Mean ± SD, *n* = 5; *, *p* < 0.05; **, *p* < 0.01; #, 0.05 < *p* < 0.1. control, normal control; HLU, hindlimb unloading; HLU + agomir (mimic), hindlimb unloading + transfected novel-miRNA agomir; HLU + antagomir (inhibitor), hindlimb unloading + transfected novel-miRNA antagomir; BV/TV, bone volume fraction.

After 28 days of HLU, the femoral mass in both the HLU and HLU + novel-miRNA agomir groups was lower compared to the control group (*p* < 0.05). The femoral mass was numerically higher in the HLU + novel-miRNA antagomir group than that in the HLU and HLU + agomir groups, with a marginal difference (0.05 < *p* < 0.1, Figure 6B). Compared with the control group, the BV/TV in the HLU and HLU + novel-miRNA agomir groups were lower (*p* < 0.05). The HLU + novel-miRNA antagomir group exhibited a 15.01% numerical increase in BV/TV relative to the HLU group, though the difference did not reach statistical significance (0.05 < *p* < 0.1, Cohen’s d = 0.71, 95% CI [−0.16, 1.38], Figure 6D). Masson staining revealed a significant reduction in positively stained area in the HLU and HLU + novel-miRNA agomir groups compared to the control group (*p* < 0.05). Relative to the HLU group, agomir treatment led to a 15.88% numerical reduction in the positively stained area, showing a marginal statistical difference (0.05 < *p* < 0.1, Cohen’s d = 0.69, 95% CI [−0.13, 1.41]), whereas antagomir treatment significantly increased the positively stained area by 27.13% (*p* < 0.05, Figure 6G). Expression of the novel miRNA in serum-derived sEVs was significantly elevated in the HLU group compared to the control group (*p* < 0.05) and was further significantly upregulated in the HLU + novel-miRNA agomir group relative to both the control and HLU groups (*p* < 0.01, Figure 6F).

## 4. Discussion

Quantitative and morphological analyses of musculoskeletal tissues across hibernation stages in Daurian ground squirrels revealed only minimal, non-significant declines in soleus muscle mass and myofiber cross-sectional area (*p* > 0.05). These observations align with earlier findings [3,28], supporting the capacity of this species to preserve musculoskeletal integrity during prolonged inactivity. Other hibernating rodents demonstrate similar resistance to disuse-induced musculoskeletal deterioration [29]. For example, golden-mantled ground squirrels maintain soleus muscle mass throughout hibernation [30], and woodchucks (Marmota monax) show no significant loss in cortical bone density, bone volume fraction, and trabecular microstructure [31]. Likewise, bone architecture in the hindlimbs of Arctic ground squirrels (*Urocitellus parryii*) and golden-mantled ground squirrels remains well preserved during prolonged torpor [5,32]. In contrast, non-hibernating rodents subjected to 14 days of HLU display pronounced musculoskeletal degradation, including a 50% reduction in myofiber cross-sectional area, a 33–50% decrease in soleus muscle mass [33,34], and concomitant bone deterioration. Notably, both hibernating and non-hibernating animals exhibit coordinated musculoskeletal remodeling under conditions of reduced mechanical loading, but hibernators uniquely resist disuse-induced atrophy and structural loss. This adaptive phenotype highlights Daurian ground squirrels as a powerful natural model for studying endogenous resistance to skeletal muscle wasting and osteoporosis.

Recent research has expanded the understanding of muscle–bone communication beyond mechanical signaling, highlighting the significance of biochemical interactions. Among these, exosomes—classified as a subtype of sEVs—have emerged as key mediators of intercellular signaling through autocrine, paracrine, and endocrine mechanisms [15,16,17,35]. The results of this study confirm that Mu-EVs were efficiently internalized by preosteoblasts, with predominant perinuclear localization. This observation is consistent with prior reports demonstrating that fluorescently labeled sEVs derived from C2C12 myoblasts, following systemic administration in mice, migrate to multiple organs, including lung, liver, spleen, brain, heart, pancreas, and gastrointestinal tissues [36]. In functional assays, Mu-EVs isolated from both PRE and TOR stages significantly upregulated the expression of osteogenic markers, including ALP, OCN, and OPN, in MC3T3-E1 preosteoblasts. These data support a role for Mu-EVs in promoting osteogenic differentiation and remodeling and suggest that sEV-mediated signaling may serve as a crucial mechanism in the maintenance and reconstruction of muscle–bone crosstalk. Importantly, under identical treatment conditions, TOR-Mu-EVs from squirrels induced significantly higher expression of mid-to-late osteogenic markers than PRE-Mu-EVs. This discrepancy suggests that hibernating squirrels may remodel the cargo composition of Mu-EVs to potentiate osteoinductive signaling at the late osteogenesis stage, which could contribute to the mitigation of disuse-associated bone loss during prolonged inactive.

Studies have demonstrated that the miRNA cargo of sEVs differs substantially from that of their parental cells [37,38] and exhibits dynamic modulation in response to physiological or pathological cues. For instance, sEVs derived from the atrophic gastrocnemius of streptozotocin-induced diabetic rats show reduced levels of miR-23a, whereas dexamethasone stimulation of C2C12 myoblasts increases miR-23a abundance in secreted sEVs [39]. Conversely, sEVs released by senescence-associated atrophic skeletal muscle cells are highly enriched in miR-29b-3p and suppress neuronal differentiation, suggesting that senescent muscle may disrupt neuromuscular signaling by altering sEV miRNA content [40]. In the present study, Mu-EVs promoted osteogenic differentiation, with a more pronounced effect observed following torpor. This enhancement likely reflects adaptive changes in miRNA composition induced by prolonged inactivity during hibernation. Supporting this, skeletal muscle disuse following sciatic nerve transection in mice has been shown to trigger significant upregulation of miR-206 and downregulation of miR-1, miR-133a, and miR-133b within Mu-EVs [41].

Comprehensive miRNA profiling of Mu-EVs from PRE and TOR groups identified 41 differentially expressed miRNAs, including six upregulated and 35 downregulated species in TOR-Mu-EVs compared with PRE-Mu-EVs. This global reduction in miRNA cargo during torpor reflects the hypometabolic state characteristic of hibernation, marked by suppressed transcriptional and energetic activity. GO enrichment analysis of predicted miRNA targets revealed significant enrichment in various biological processes including the cellular process, metabolic process, and biological regulation; cellular components such as the cell, cell part, and organelle; and molecular functions involving binding and catalytic activity. These patterns indicate that differentially expressed miRNAs regulate key aspects of cellular metabolism, structure, and enzymatic function. KEGG pathway analysis of target genes identified several enriched signaling pathways, including the TGF-β signaling pathway, MAPK signaling pathway, ECM–receptor interaction, and ether lipid metabolism implicated in cell communication, plasticity, and metabolism. Given the well-preserved musculoskeletal architecture observed during hibernation, these findings implicate TOR-Mu-EVs in modulating musculoskeletal maintenance under conditions of prolonged inactivity. This hypothesis aligns with prior work showing that Mu-EV-associated small RNAs regulate signaling pathways essential for preserving muscle mass, cytoskeletal organization, and neuromuscular connectivity [42]. Research has also demonstrated that Mu-EVs influence osteoblast function and bone remodeling by orchestrating processes related to energy metabolism, tissue repair, and immune modulation [43].

Hibernating ground squirrels, which naturally resist disuse-induced osteoporosis, provide a unique model for identifying regulatory pathways involved in skeletal maintenance under prolonged inactivity. Pathway enrichment analysis demonstrated that target genes of differentially expressed miRNAs are enriched in the TGF-β signaling cascade. BMP7, an important member of this pathway, is a central regulator governing osteoblast differentiation [44]. These findings suggest that TOR-Mu-EVs may influence bone metabolism by modulating BMP7 expression. Consistently, qPCR and dual-luciferase reporter assays confirmed that a novel miRNA, significantly downregulated in the TOR group, physically binds to the BMP7 3′UTR. Functional assays in MC3T3-E1 cells demonstrated that overexpression of this miRNA suppressed BMP7 and downstream Smad1/5/8 expression, thereby attenuating osteogenic differentiation, as reflected by reduced expression of osteogenic markers. In contrast, miRNA inhibition enhanced BMP7 and phosphorylated Smad1/5/8 (p-Smad1/5/8) levels, promoting osteogenic differentiation. These results indicate that the hibernation-associated, Mu-EV-mediated downregulation of this miRNA may relieve repression of BMP7 and facilitate osteogenesis via the Smad1/5/8 pathway. Furthermore, the observed reduction in the level of this miRNA in TOR-Mu-EVs suggests a mechanism by which muscle-derived sEVs help maintain bone homeostasis by modulating BMP7 signaling. Consistent with these results, prior studies have demonstrated that inhibition of miR-342-5p upregulates BMP7 expression and promotes osteoblast proliferation and differentiation [45], while BMP7 has been shown to enhance OCN and OPN expression through Smad1/5/8 activation, thereby promoting osteogenic differentiation and maintaining bone homeostasis [46]. Additional evidence indicates that sEVs from C2C12 myoblasts promote MC3T3-E1 osteogenic differentiation via miR-27a-3p-mediated activation of the β-catenin pathway [18] and inhibit osteoclastogenesis through high miR-196a-5p content [20]. Collectively, these findings confirm that the sEV-mediated miRNA pathway serves as a crucial messenger in bone maintenance and remodeling, exerting a positive regulatory role in bone metabolism by promoting bone formation and inhibiting bone resorption. Notably, we observed a reduction in overall sEV production, along with diminished loading of anti-osteogenic miRNAs per vesicle. We speculate that cells undergo cargo remodeling of sEVs under treatment. This remodeling acts synergistically via two distinct pathways, suppressed total vesicle secretion and selective down-sorting of osteoinhibitory miRNAs, which jointly mitigate inhibitory osteogenic signals and maintain bone structural homeostasis.

In vivo transfection experiments demonstrated that mechanical unloading and miRNA agomir exacerbate disuse-induced skeletal loss in mice, whereas miRNA antagomir transfection partially attenuates unloading-associated bone impairment. Consistent with this pattern, Masson staining showed a 15.88% numerical reduction in positively stained area in the HLU + agomir group relative to HLU alone (0.05 < *p* < 0.1), while a significant increase was observed in the HLU + antagomir group (*p* < 0.05). These findings suggest that the novel miRNA may exert a partial regulatory effect on new bone deposition. Micro-CT analysis further revealed a reduction in BV/TV in the HLU + agomir group compared with the control group, whereas no significant difference was detected following antagomir treatment. Together, these data indicate that intramuscular modulation of novel-miRNA signaling influences skeletal responses under unloading conditions, although the overall impact on bone formation remains modest. This limited effect may reflect insufficient in vivo transfection efficiency, tissue-specific differences in extracellular vesicle uptake, and the intrinsic property of the miRNA–Bmp7 interaction: their binding site falls into the lowest-confidence class of canonical 6mer seed pairing. Such sites typically mediate mild repressive effects. In addition, the pleiotropic nature of miRNA regulation suggests that, beyond BMP7, this novel miRNA likely engages additional targets and signaling pathways, underscoring the complexity of sEV-mediated regulatory networks in organismal physiology.

Quantitative PCR analysis of serum sEVs from HLU mice demonstrated significantly elevated levels of the novel miRNA in the HLU group compared to normal control group. Integrated dual-luciferase reporter and cell transfection assays indicated potential involvement of this miRNA in the pathogenesis of disuse osteoporosis through suppression of BMP7 expression. Importantly, pathological unloading and adaptive torpor exert opposing regulatory effects on sEV–miRNA axis-mediated skeletal homeostasis, as evidenced by the downregulation of this miRNA in the TOR group. This inverse relationship forms the core conceptual framework of the present study. Furthermore, serum sEV levels of the novel miRNA were further increased in the HLU + agomir group compared to the HLU group alone. In conjunction with in vivo transfection results, this pattern supports delivery of the exogenous miRNA to hindlimb skeletal muscle, followed by release into the systemic circulation via the sEVs pathway, thereby influencing bone formation at distal sites. Similar muscle-to-organ communication has been reported previously, including adeno-associated virus (AAV)-mediated overexpression of miR-23a and miR-27a in skeletal muscle, which attenuates diabetes-associated renal fibrosis through muscle-kidney signaling mediated by Mu-EVs [47], as well as intramuscular delivery of miR-26, which alleviates chronic kidney disease-associated myocardial fibrosis through sEV-dependent mechanisms [48]. Collectively, these observations support a role for muscle-derived sEVs as systemic carriers of regulatory miRNAs that modulate pathological processes in remote tissues.

## 5. Conclusions

In summary, this study provides a systematic characterization of miRNA dynamics within Mu-EVs, associated signaling pathways, and regulatory mechanisms governing bone metabolism during prolonged inactivity in hibernating animals. Mu-EVs derived from hibernating ground squirrels enhanced osteogenic responses through sEV-mediated signaling. Moreover, the downregulation of a novel miRNA within TOR-Mu-EVs alleviated suppression of BMP7 and likely promoted osteogenic differentiation through activation of Smad1/5/8 signaling, thereby contributing to the maintenance of bone homeostasis during extended hibernation-associated disuse. Notably, pathological unloading and adaptive torpor exert opposing regulatory effects on skeletal homeostasis governed by the sEVs–miRNA axis. These findings suggest that sEV-associated miRNA signaling constitutes a potential mechanism uncovered in hibernating squirrels. This pathway enables partial protection against disuse osteoporosis and yields candidate molecular targets for therapeutic development.

## 6. Limitations

This study found that Mu-EVs derived from hibernating ground squirrels enhanced the osteogenic response through EV-mediated signaling. However, it remains to be further investigated how reduced EV abundance is reconciled with preserved musculoskeletal integrity, whether qualitative changes in sEV cargo may outweigh quantitative changes, and whether sEV function, rather than sEV number, is the primary determinant of the observed effects. Additionally, the mutant Bmp7 luciferase reporter used in this work disrupts the full predicted miRNA–mRNA duplex rather than introducing selective mutations restricted solely to the seed region. Accordingly, the existing luciferase assay results cannot unambiguously discriminate canonical seed-dependent miRNA regulation from effects mediated by extensive siRNA-like base pairing. Follow-up experiments using reporter constructs harboring seed-region-only mutations are needed to independently validate seed-dependent interactions. Furthermore, the relatively small sample size in the present study may limit statistical power. Larger animal cohorts will be recruited in follow-up investigations to systematically characterize hibernation-associated alterations in bone microarchitecture. Finally, we lack protein-level Western blot validation of osteogenic markers to support transcript-level results. Subsequent studies will perform Western blot on Mu-EV-treated MC3T3-E1 cells to validate TOR-Mu-EV pro-osteogenic activity and strengthen evidence for the sEV–miRNA regulatory axis in bone homeostasis.

## Data Availability

The data that support the findings of this study are available from the corresponding authors upon reasonable request.

## Supplementary Materials

The following supporting information can be downloaded at: https://www.mdpi.com/article/10.3390/cells15161468/s1, Figure S1: State of Daurian ground squirrels in different periods; Figure S2: Western blotting image of miRNA transfection on osteogenesis of MC3T3-E1 cells; Figure S3: Protein markers for characterizing small extracellular vesicles; Figure S4: miRNA structure; Table S1: Animal groups and sample time; Table S2: Antibody information; Table S3: Primers of genes; Table S4: List of miRNA primers; Table S5: Plasmid transfection combinations information; Table S6: Related miRNA gene sequences; Table S7: Related antibody information.
