# Supplementary material for: Muscle-Derived Small Extracellular Vesicles Regulate Bone Maintenance During Hibernation Through miRNA-Mediated Signaling
Source: Cells. 2026 Aug 16;15(16):1468. doi: 10.3390/cells15161468 (PMC13511335; doi:10.3390/cells15161468)
Supplement: Supplementary file 1 [file cells-15-01468-s001.zip › supplementary material 1.pdf]

Supplementary Material 1：

Figure S1. State of Daurian ground squirrels in different periods

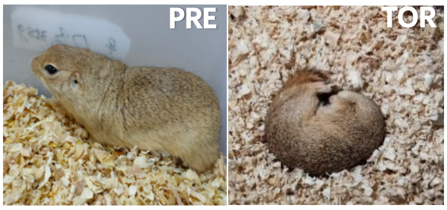

PRE, pre-hibernation group; TOR, torpor group.

Table S1. Animal groups and sample time

Group	Sample time	Tb of animal
PRE	Mid-October	>37 °C
TOR	After two months hibernation (Mid-late January of the following year)	≥5 days with stable Tb of 5–8 °C

Notes: PRE, pre-hibernation group; TOR, torpor group.

Table S2. Antibody information

Antibody name	Dilution rate	Product code	Manufacturers
CD63	1:1000	YT5525	ImmunoWay
TSG101	1:2000	28283-1-AP	PROTEINTECH
HSP70	1:5000	10995-1-AP	PROTEINTECH
Calnexin	1:1000	YT0613	ImmunoWay
Goat anti Rabbit IgG-HRP	1:10000	111-035-003	Jackson ImmunoResearch

Table S3. Primers of genes

Gene name	Primer sequence
ALP	Forward: GGAATACGAACTGGATGAGAAGG<br>Reverse: GGTTCCAGACATAGTGGGAATG
OPN	Forward: GATTCTGTGGACTCGGATGAAT<br>Reverse: GTAGGGACGATTGGAGTGAAAG
OCN	Forward: GGAGGGCAATAAGGTAGTGAAC<br>Reverse: CAAGCCATACTGGTCTGATAGC
RUNX2	Forward: CCAAGAAGGCACAGACAGAA<br>Reverse: CGGGACACCTACTCTCATACT
BMP7	Forward: GGGAGTTCCGGTTTGATCTT<br>Reverse: GATCTGGAAGGTCTCTTGTC
β-actin	Forward: GTCCCTCACCCTCCCAAAAG<br>Reverse: GCTgCCTCAACACCTCAACCC

Table S4. List of miRNA primers

Gene name	Primer sequence (5`-3`)
novel-miRNA-3p RT	GTCGTATCCAGTGCAGGGTCCGAGGTATTCGCAC<br>TGGATACGACAGGAG
novel-miRNA-3p	Forward: AGGCAGCAGCCCGGCTCTC<br>Reverse: GTGCAGGGTCCGAGGTATTC

U6	Forward: CTCGCTTCGGCAGCACA<br>Reverse: AACGCTTCACGAATTTGCGT

Table S5. Plasmid transfection combinations information

Number	Transfection plasmid combination
1	NC-mimics + BMP7-WT
2	novel-miRNA-mimics + BMP7-WT
3	NC-mimics + BMP7-MT
4	novel-miRNA-mimics + BMP7-MT

Table S6. Related miRNA gene sequences

Name	Gene sequence (5`-3`)
novel-miRNA agomiR	GCAGCAGCCCGGCTCTCCTAAT
novel-miRNA antagomiR	ATTAGGAGAGCCGGGCUGCUGC
novel-miRNA NC	CAGUACUUUUGUGUAGUACAA

Table S7. Related antibody information

Antibody name	Dilution rate	Product code	Manufacturers
ALP	1:500	sc-365765	Santa
RUNX2	1:1000	YT5356	ImmunoWay
OCN	1:500	sc-365797	Santa
OPN	1:1000	sc-21742	Santa
BMP7	1:1000	YT0503	ImmunoWay
Smad1/5/8	1:1000	#21684	Signalway
p-Smad1/5/8	1:500	AB3848-I	Sigma-Aldrich
HRP Goat Anti-mouse IgG (H+L)	1:2000	AS003	Abclonal
HRP Goat Anti-rabbit IgG (H+L)	1:2000	AS014	Abclonal
