# Supplementary material for: Muscle-Derived Small Extracellular Vesicles Regulate Bone Maintenance During Hibernation Through miRNA-Mediated Signaling
Source: Cells. 2026 Aug 16;15(16):1468. doi: 10.3390/cells15161468 (PMC13511335; doi:10.3390/cells15161468)

Figure S2. Western blotting image of miRNA transfection on osteogenesis of MC3T3-E1 cells (n = 3). (A) BMP7, (B) OCN, (C) OPN, (D) Runx2, (E) Smad1/5/8. Notes: BMP7, Bone morphogenetic protein 7; OCN, Osteocalcin; OPN, Osteopontin; Runx2, Runt-related transcription factor 2. 1, blank control group; 2, negative control group; 3, novel-miRNA antagomir group (inhibitor group); 4, novel-miRNA agomir group (mimics group). Blank control group (1) values were used as a normalization control for multiple plates.

(A)BMP7 (49Kda)

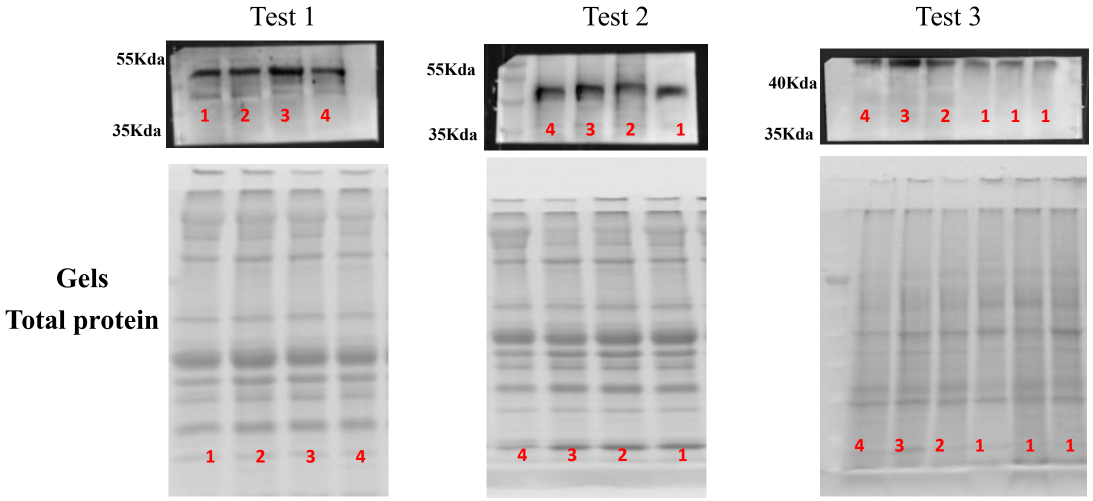

(B)OCN (15Kda)

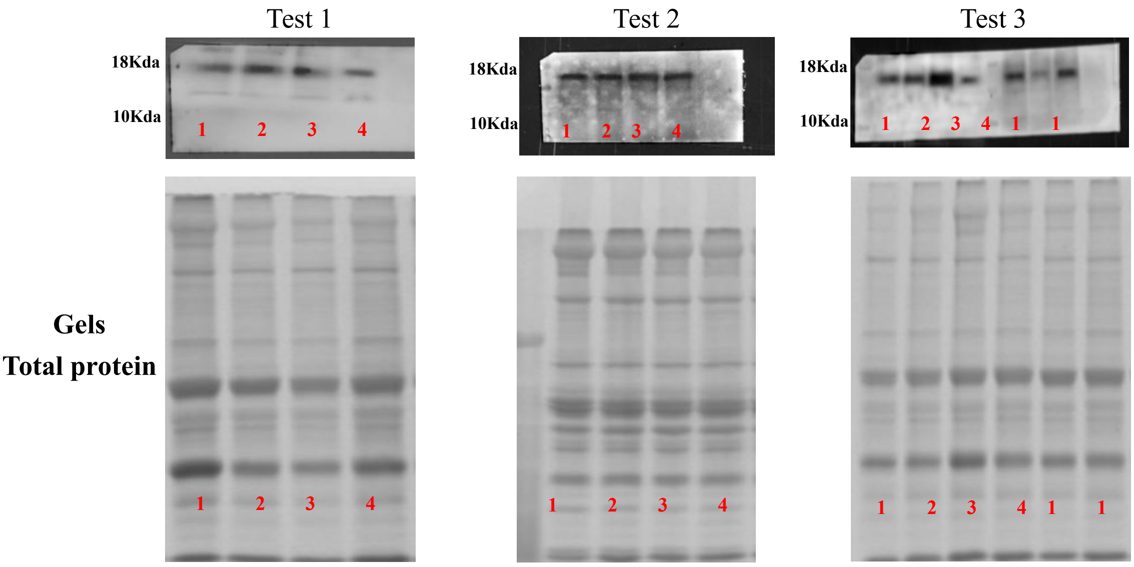
Test 1
Test 2
Test 3
18Kda
10Kda
1 2 3 4
1 2 3 4
1 2 3 4 1 1
Gels
Total protein
1 2 3 4
1 2 3 4
1 2 3 4 1 1

(C) OPN (55Kda)

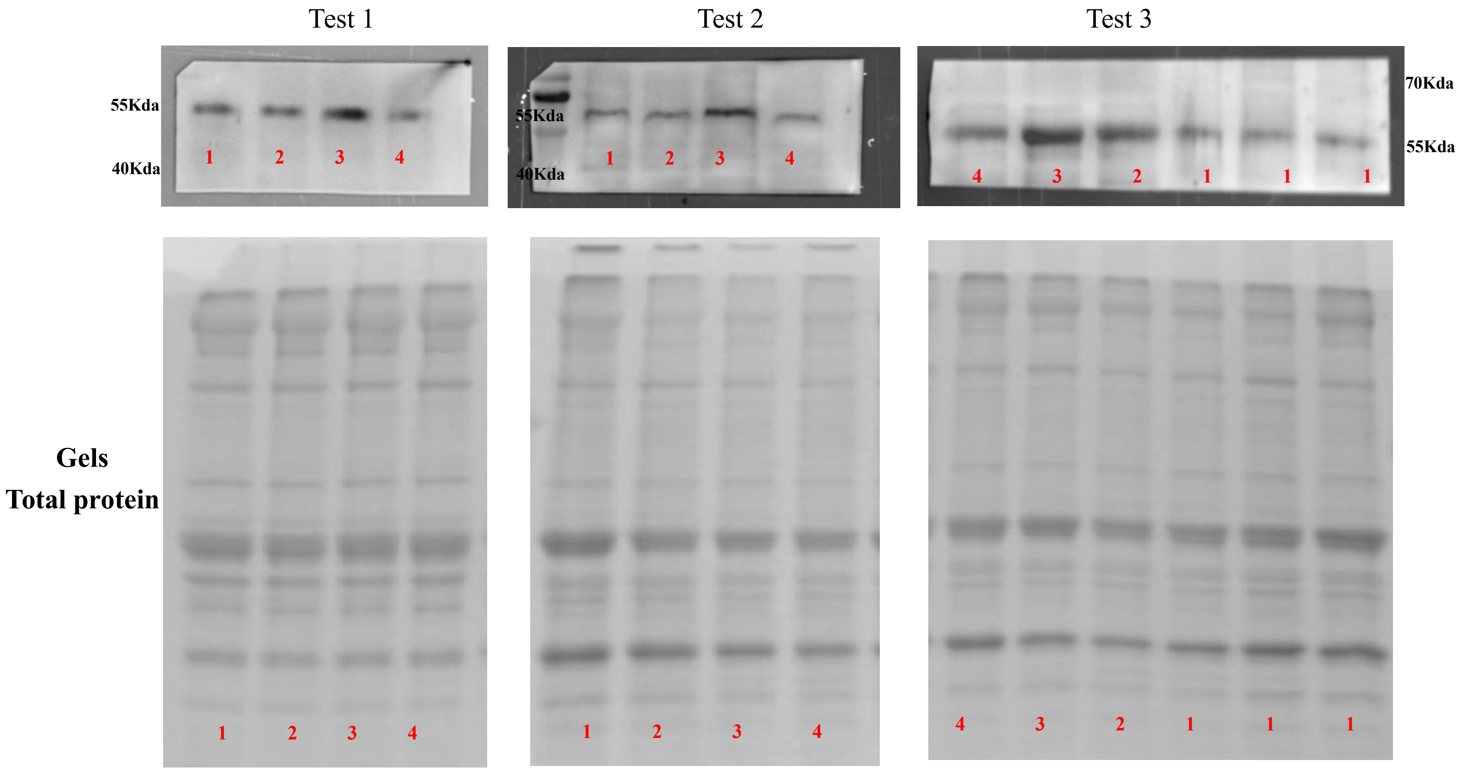

Test 1
Test 2
Test 3
55Kda
40Kda
1 2 3 4
55Kda
40Kda
1 2 3 4
70Kda
55Kda
4 3 2 1 1 1
Gels
Total protein
1 2 3 4
1 2 3 4
4 3 2 1 1 1

(D) Runx2 (57Kda)

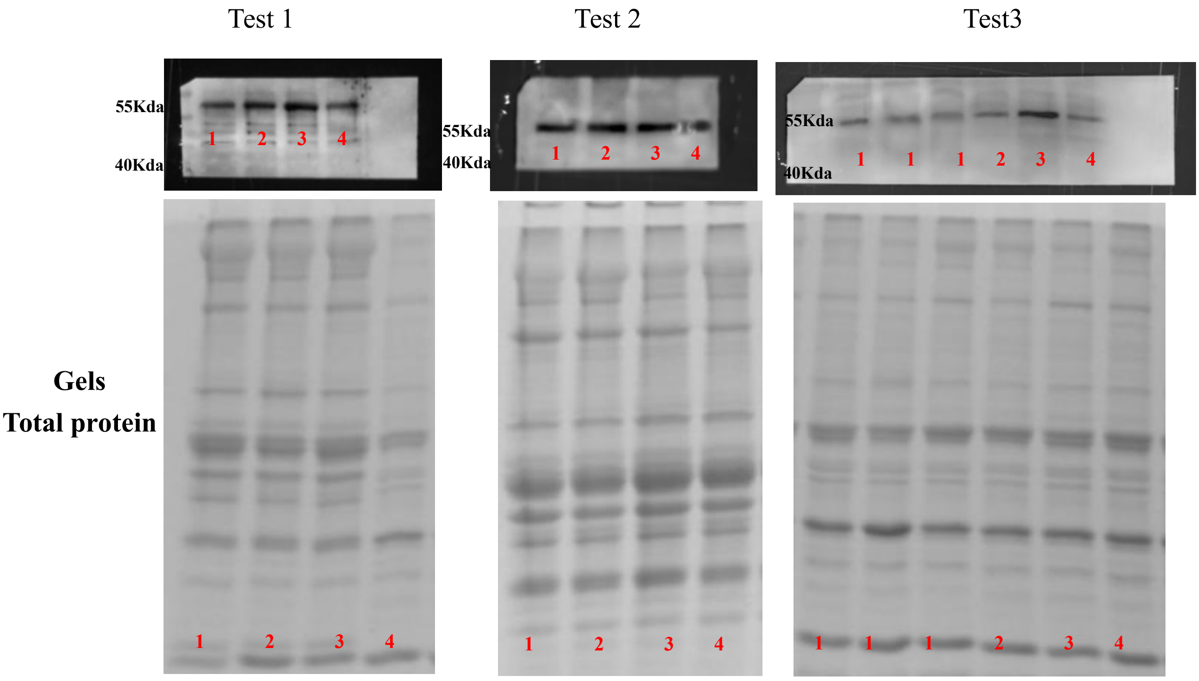

(E) Smad 1/5/8 (50Kda)

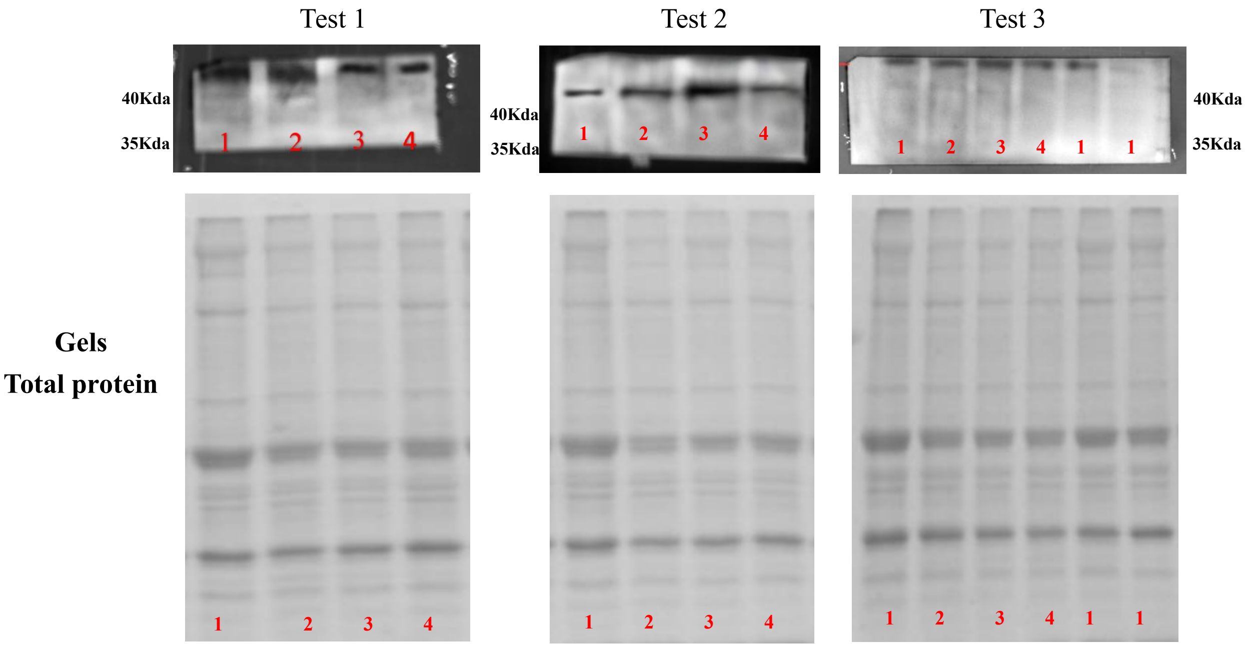

Figure S3. Protein markers for characterizing small extracellular vesicles. (A) TSG101, (B) HSP70, (C) CD63, (D) Calnexin, (E) Calnexin (positive control)

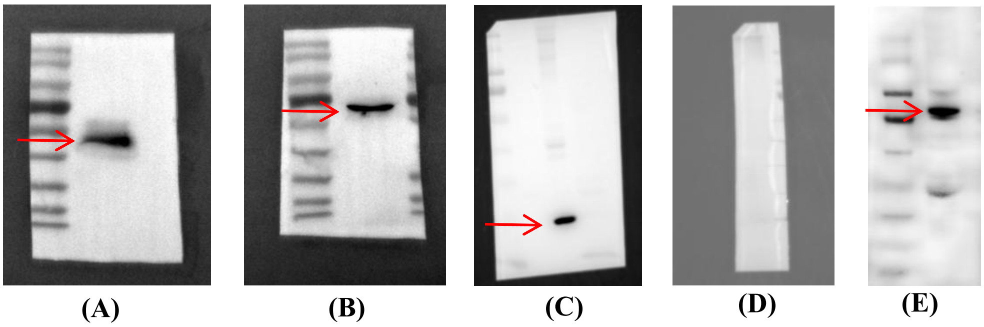

Figure S4. miRNA structure (A) total score = 16, star read score = 3.9, read count score = 10.3, mature read count = 31, star read count = 1, TargetScan Custom Predictor（B） and Freiburg RNA Tools miRNA-Bmp7 target prediction (C).

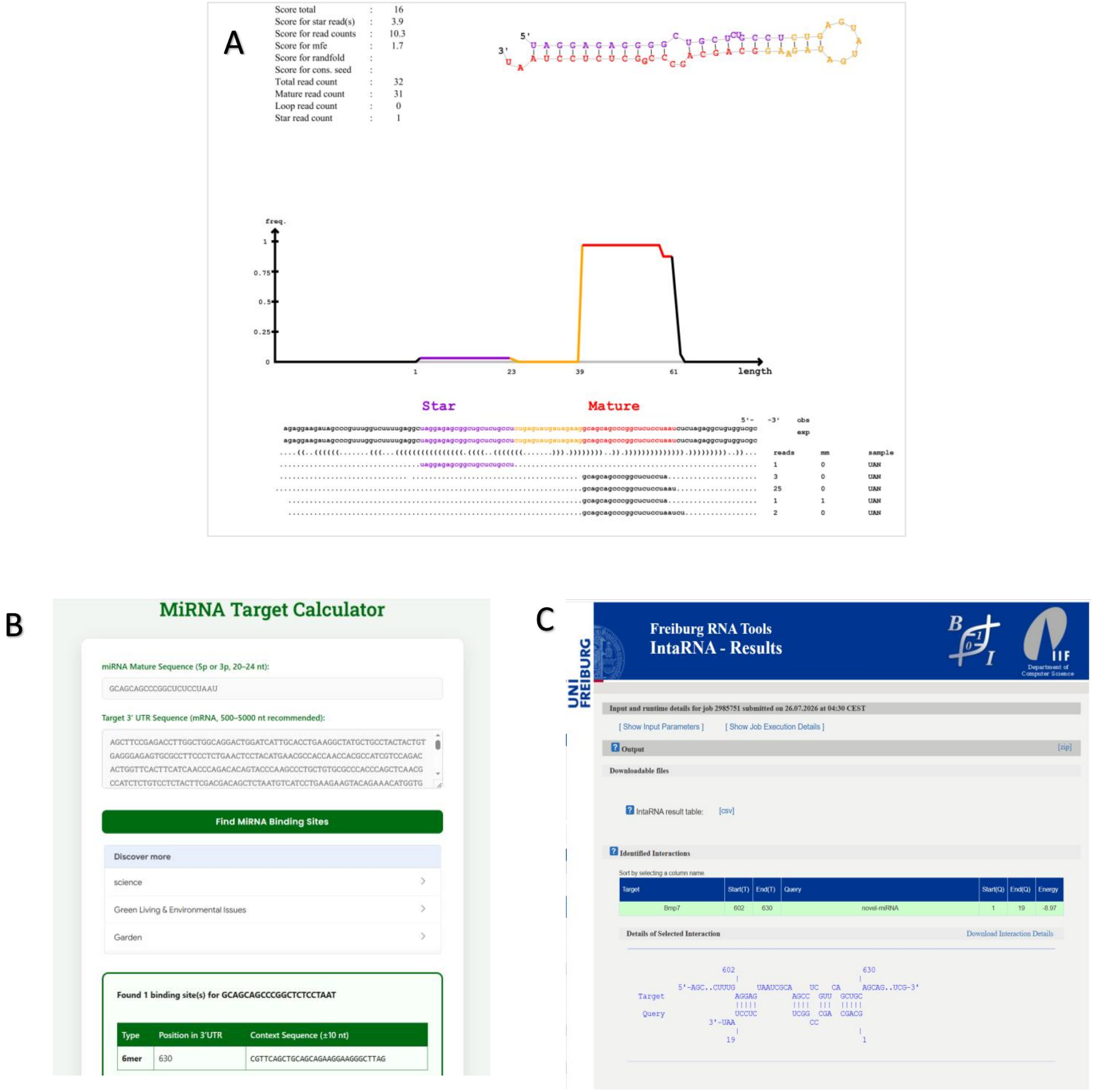

Supplement: Supplementary file 1 [file cells-15-01468-s001.zip › Supplementary Material 2 .pdf]
